# Intersectional analysis of visual generative AI: the case of stable diffusion

**DOI:** 10.1007/s00146-025-02207-y

**Published:** 2025-03-17

**Authors:** Petra Jääskeläinen, Nickhil Kumar Sharma, Helen Pallett, Cecilia Åsberg

**Affiliations:** 1 Division of Media Technology and Interaction Design, Department of Human-Centered Technology, EECS, KTH Royal Institute of Technology, Stockholm, Sweden; 2 3S (Science, Society & Sustainability) Research Group, School of Environmental Sciences, University of East Anglia, Norwich Research Park, United Kingdom; 3 Department of Data Science, School of Interwoven Arts and Sciences, Krea University, Sri City, Andhra Pradesh India; 4 Department of Thematic Studies: Gender Studies, Linköping University, Linköping, Sweden

**Keywords:** Critical AI, Stable diffusion, Generative AI, Algorithmic reparation, Restorative justice, Intersectionality

## Abstract

Since 2022, Visual Generative AI (vGenAI) tools have experienced rapid adoption and garnered widespread acclaim for their ability to produce high-quality images with convincing photorealistic representations. These technologies mirror society’s prevailing visual politics in a mediated form, and actively contribute to the perpetuation of deeply ingrained assumptions, categories, values, and aesthetic representations. In this paper, we critically analyze Stable Diffusion (SD), a widely used open-source vGenAI tool, through visual and intersectional analysis. Our analysis covers; *(1) the aesthetics of the AI-generated visual material, (2) the institutional contexts in which these images are situated and produced, and (3) the intersections between power systems such as racism, colonialism, and capitalism*—which are both reflected and perpetuated through the visual aesthetics. Our visual analysis of 180 SD-generated images deliberately sought to produce representations along different lines of privilege and disadvantage—such as wealth/poverty or citizen/immigrant—drawing from feminist science and technology studies, visual media studies, and intersectional critical theory. We demonstrate how imagery produced through SD perpetuates pre-existing power systems such as sexism, racism, heteronormativity, and ableism, and assumes a default individual as white, able-bodied, and masculine-presenting. Furthermore, we problematize the hegemonic cultural values in the imagery that can be traced to the institutional context of these tools, particularly in the tendency towards Euro- and North America-centric cultural representations. Finally, we find that the power systems around SD result in the continual reproduction of harmful and violent imagery through technology, challenging the oft-underlying notion that vGenAI is culturally and aesthetically neutral. Based on the harms identified through our qualitative, interpretative analysis, we bring forth a reparative and social justice-oriented approach to vGenAI—including the need for acknowledging and rendering visible the cultural-aesthetic politics of this technology and engaging in reparative approaches that aim to symbolically and materially mend injustices enacted against social groups.

## Introduction

Stable Diffusion (SD) by StabilityAI was launched in August 2022, and it swiftly gained 10 million monthly users (Stability AI 2023). From there on, Visual Generative AI (vGenAI) technologies have become commonplace in everyday use, with audiovisual generative AI having an estimated market of $8.28 billion USD in 2022, projected to grow up to $99.79 billion USD by 2030 (Virtue Market Research 2024). So far, SD has generated more than *12 billion images*—for context, human photographers took 150 years to generate 15 billion images—with 10 million users generating up to 2 million images every day (Everypixel Journal 2024). Aligned with this development, vGenAI tools, such as SD, DALL-E, and Midjourney, have also quickly become widely used in the design and creative fields (Totlani 2023; Van Der Maden et al. 2023).

Visual GenAI (vGenAI) is a subset of broader generative AI (GenAI) technologies^1^, and together they face significant scrutiny and critique. These critiques include a lack of transparency and fairness (Ray 2023; Bender et al. 2021), accountability (Weidinger et al. 2022), data protection and privacy (Wang et al. 2023; Golda et al. 2024), environmental sustainability (Jääskeläinen et al. 2022), implications for human creativity (Arielli et al. 2022), copyright infringement and misuse of creatives’ work (Samuelson 2023), gender and ethnicity-based discrimination (Almeida et al. 2024; D’Ignazio and Klein 2020), and the generation and spread of misinformation, such as the viral 'Balenciaga Pope' deepfake image (Perrigo 2023). vGenAI, in particular, faces additional criticism related to the inclusion of harmful images in training data (Birhane et al. 2021). Meanwhile, regulators, lawyers, ethics committees, copyright holders, and creatives whose ability to make a living is threatened by these technologies, have struggled to catch up with and adequately respond to the implications of vGenAI. The swift and extensive adoption of vGenAI, coupled with existing concerns in public discourse and academia, underscores the need for critical examination and careful analysis of its images, design, and socio-technical systems around it.

Addressing this need, our paper puts forward a visual cultural, and intersectional analysis of a specific vGenAI system, Stable Diffusion XL (SDXL). By grounding our analysis in visual material of 180 images generated using SD, we respond to recent calls in ethics of GenAI (Hagendorff 2024) to provide evidence grounded in a particular technology and context. The paper addresses three research questions: *(i) how are different social categories represented aesthetically in the imagery? (ii) how do different institutions come together to further marginalize certain social categories*? and *(iii) how do various power systems (e.g., sexism, colonialism, capitalism) intersect and come together as vehicles of oppression?* Our methodological approach draws primarily from feminist Science and Technology Studies (STS) (e.g. Wajcman 2010; D’Ignazio and Klein 2020; Adkins et al. 1999; Lury 2020), intersectional critical theory (Crenshaw 1989; Sharma et al. 2023), and feminist media and visual cultural studies (Evans 1999; Hall 1997). We perform this analysis on three levels; *(1) micro-level by examining the aesthetics of the AI imagery*, *(2) meso-level by examining the institutional contexts in which the images are situated and produced,* and *(3) macro-level by analyzing the intersections between power systems (such as racism, colonialism and capitalism).* In our analysis, the primary qualitative material of AI imagery is complemented through a review of online sources and literature that helps to make sense of the systemic embeddedness of SDXL. The qualitative visual analysis is performed using an interpretative approach (e.g. Gillian 2012) that involves textually documenting, describing, reflecting on, and analyzing the imagery and its aesthetics.

We organize and present our image analysis in three themes; *(1) visual representations of societal privilege and discrimination, (2) visual ideological representations,* and *(3) visual representations of people in everyday scenarios in different cultural contexts*. These descriptions are then brought into further intersectional analysis. By critiquing the images, the technology that generated them, and the socio-technical context around the technology, for the first time, we examine how privilege and discrimination manifest at the micro (individual), meso (institutional), and macro (power systems) levels. Through this, we urge reflection on embedding anti-oppressive, anti-structural forces in technology design, and suggest potential ways forward through an intersectional feminist approach requiring dialogue between academia, industry, creative practitioners, and policymakers. Specifically, we demonstrate how imagery produced through SD perpetuates pre-existing marginalization (e.g. sexism, racism, heteronormativity, and ableism) while assuming a default figure as white, able-bodied, and masculine-presenting. We trace these power dynamics embedded and inherent in the imagery back to the institutional and cultural context of the technology, particularly Euro- and North America-centric cultural representations. We argue that the power systems surrounding this technology result in the active and continual reproduction of harmful and violent imagery through the technology. Based on the harms brought forth by our analysis, we suggest a reparative and social justice-oriented approach to vGenAI as a path forward, discuss the need for acknowledging the cultural-aesthetic politics of vGenAI, and suggest concrete pathways (such as assigning criminal culpability for AI imagery-related harms) moving forward.

## Towards an intersectional framework to analyze vGenAI

In this section we describe our visual cultural and intersectional theoretical framework. We first (2.1) begin by covering the societal critique that relates to vGenAI, and discuss the theoretical underpinnings of visual culture in relation to vGenAI. In the second Sect. (2.2), we present the three dimensions of our intersectional analysis framework.

### Visual and cultural perspectives on vGenAI

Benjamin (2019) elucidates how digital technologies often mirror and perpetuate existing inequalities, despite being heralded as objective or progressive compared to previous discriminatory systems. The modernist and rationalist pursuit of objectivity, efficiency, profitability, and progress, often underlies these technical endeavors. There is a large body of work that takes similar feminist and critical lenses on AI technologies generally (e.g. Buolamwini 2019; Danielescu 2020; Kapania et al. 2023; Scheuerman et al. 2020; Sparrow 2020; Dray et al. 2014; Bardzell et al. 2011; Snyder 2020; Crawford 2021; Bender et al. 2021), and vGenAI specifically (Luccioni et al. 2023; Offert et al. 2022; Jääskeläinen et al. 2024; Almeida et al. 2024; Anand et al. 2023; Sun et al. 2024). Currently, numerous analyses have addressed the negative stereotyping perpetuated by vGenAI. For instance, a study by Gorska and Jemielniak (2023) revealed that when prompting AI image generators for certain professions such as CEO, heteronormative masculine representations^2^ were significantly overrepresented, while heteronormative feminine representations were overrepresented in lower-paying positions, such as primary school teachers. Similarly, an independent analysis by journalists at Bloomberg found that heteronormative masculine representations with lighter skin tones dominated subjects in high-paying jobs (Leonardo and Dina 2023). At the same time studies, such as Clemmer et al. (2024) have aimed to technically address the limitations of these systems. As the applications of these technologies expand into various domains of visual media (Jiang et al. 2023), including virtual reality, AI-generated film, video games, deep-fake political propaganda, and revenge porn, as well as commercial culture, advertising, and digital artwork, it is crucial to interrogate the differential impacts that visual cultural politics can have on various social groups.

However, there is a lack of multi-factor and integrative analyses particularly from an intersectional perspective, which bring together different identity categories and social groups in the analysis while relying on strong empirical visual material. Most existing analyses focus on isolating the various dimensions of intersectional analysis, e.g. focusing explicitly on gender or race, and overlooking the systemic embeddedness of the technology. While to date there has been no empirical understanding of the harms perpetuated by SDXL, it is clear that the access to and the potential benefits and harms of vGenAI technology are not equitably distributed across all social groups, such as users with special needs, or users in the Global South. This is due to the unequal distribution of resources, unequal access, and differing cultural and political representation that reinforces the existing forms of oppression (Ciurria 2023; Arora 2016; Tacheva and Ramasubramanian 2023). Furthermore, the power systems of gender, sexuality, class, race, and colonialism are present in data that is used to train vGenAI, calling for feminist interventions on pronounced representational concerns in the emerging media ecologies of visual culture (Jääskeläinen 2024; Sun 2024). A further socio-technical critique that highlights the mechanisms of discrimination through visual technology is the term ‘coded gaze’, suggesting that such representations are deeply embedded within AI technologies (Buolamwini 2023). This term is derivative of feminist media studies concept of 'male gaze' (Wekker 2017), already building a connection between AI technology and visual culture that our study more explicitly addresses.

**Fig. 1:**
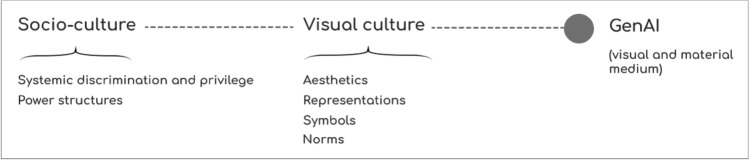
The relation between vGenAI, visual culture, and socio-culture at large

As discussed above, while gender- and ethnicity-focused analyses of AI imagery are becoming common, there is also a lack of work examining ‘visual political processes of portrayal’ (Jääskeläinen et al. 2024) that acknowledges the design political (Fry 2010; Winner 1980) and cultural political nature of vGenAI by bringing together its imagery, technology, and socio-cultural context. This type of work has been generally conducted in feminist and critical media studies, which have argued that examining visual cultural politics opens a space for questioning societal power configurations and systems through examining their materiality in visual representations (particularly Sturken et al. 2017; Evans 1999; Hall 1997). More concretely, these power configurations are maintained through the process of establishing certain visual norms, and excluding representations outside of those norms (Jääskeläinen et al. 2024). Prior visual cultural studies have underscored the active construction of visual norms and culture (Boylan 2020), which also applies to vGenAI imagery with embedded stereotypes that have consequences for certain social groups in the real world. Thus, visual cultural and media studies theories suggest that vGenAI both stems from and actively establishes visual cultural norms, which in turn position people and social groups in relation to each other by constructing privilege and disadvantage (see Fig. 1 for contextualization). While the former argument (that vGenAI stems from these norms) seems more obvious, the latter can also be anticipated by examining the history of visual culture: how each newly introduced visual technology has changed the prevailing cultural landscape, norms, and epistemology of the time. A good example is the advent of photography, and how it built on the conventions of media formats that preceded it, while also bringing a new claim that was not earlier present in the production of visual culture: the concept of photography as evidence of ‘truth’ in opposition to earlier mediums that allowed artists more agency over the representation (Sturken and Cartwright 2017). vGenAI is only the next step in a long line of visual mediums, and is expected to similarly change our culture as it constitutes part of our socio-technical reality.

In summary, although research on the fairness and discrimination of vGenAI imagery abounds, there is also a dearth of studies examining their relation to broader visual culture (Jääskeläinen and Åsberg 2024). Similarly, while attention has been directed towards the negative impacts of image generators on marginalized social groups, and image generators’ neocolonial nature, the need for detailed intersectional analyses persists. There is a need to examine how vGenAI technologies contribute to and uphold marginalization through the interlocking power systems of oppression, such as racism, ableism, and capitalism. Drawing from the strong body of literature in feminist and critical lenses on media and technology studies (Adkins et al. 1999; Bloom 1999; Cartwright 1995; Gillian 2012; Jones 2010; Lury 2020; MacClintock 1995; Mulvey 1975; Sender 2016; Wekker 2016, 2017), we embark on examining these power systems within and around SDXL. Our work in this paper both draws from such an approach by contextualizing the images in the light of historical imaging conventions, and expands the critical analysis to the institutions and power systems that these technologies are embedded in.

### Intersectional STS framework for analysis of vGenAI

The concept of ‘intersectionality’ (Crenshaw 1989) initially highlighted the compounding effects of gender and race on the injustices experienced by African-American women. It emphasizes the interplay between various social categories like gender, race, and class in shaping individuals’ lives and influencing power dynamics. Unlike a simplistic accumulation of distinct oppressions, intersectionality explores how systems of oppression intersect and mutually reinforce one another. Intersectionality has since evolved into both a theoretical framework and a practical approach, extensively utilized in critical feminist scholarship (Kennedy 2005; De Vita et al. 2016). Feminist scholars contend that the application of intersectionality can be conceptualized as an orientation, wherein scholars adopting an intersectional perspective are prompted to develop a sophisticated comprehension of social and political phenomena, eschewing reductionism to singular causes (Collins 2019). This orientation necessitates reflexivity, prompting scholars to recognize the varied nature of inequalities across diverse social contexts, thereby fostering challenging dialogues and transformative ideation (Ishkanian et al. 2019). Our intersectional approach builds on Sharma et al. (2023)’s analytical framework, which encompasses 3 key lenses of intersectionality: (1) the micro (individual) lens; (2) the meso (institutional) lens; and the macro (power systems) lens.

The first lens examines the diversity of representations of individual practices and experiences to counter the dominance of specific social categories vGenAI. It contends that even when diversity in one social category, such as gender, is achieved, there is a risk of fragmenting individuals’ experiences related to other social categories like race or physical ability (Collins 2019). Thus, it advocates for approaches facilitating the exploration of the interrelationships between all aspects of lived experience and the socio-technological structures within which they occur (Kennedy 2005). Rejecting the isolation of social categories (Crenshaw 1989), this approach enables us to examine the vGenAI technological artifacts and their underlying development processes, and the impact on individuals, acknowledging the intersecting and dynamic nature of gender, race, and class. By attending to the diversity of individual subjectivities, it recognizes inequalities as interconnected, shifting, and multifaceted, encompassing both penalties and privileges (Collins and Bilge 2020). This perspective thus challenges notions of penalties associated with certain identity markers, and privileges associated with some others, paving the way for emancipatory possibilities for vGenAI users, a dimension often overlooked in current literature (Fehr 2022). It also fosters dialogue across diverse lived experiences, which could lead to the development of a plural ethic (Huang et al. 2021) to evaluate practices, policies, and social institutions related to vGenAI.

The second lens delves into the historical lineage of institutions, including BigTech companies, policymakers, regulatory bodies, and civil society to understand how they establish and perpetuate social hierarchies and influence power dynamics (Benjamin 2019; Noble 2018). It broadens the perspective on vGenAI technologies, envisioning them not just as products of current technical, economic, and sociopolitical forces, but also as products of historical forces (Hicks 2017). By uncovering processes of institutionalization and their agents, it sheds light on the deliberate actions, broader prejudices, and assumptions about social categories embedded within institutional processes. that shape these technologies. This is achieved by tracing how powerful institutions have historically contributed to injustices such as disembedding, dispossessing, dehumanizing, colonizing, and commodifying individuals (Davis et al. 2021; Williams 2021). Consequently, it prompts analyses to focus on the institutionalization of racism, sexism, classism, and other forms of discrimination, prompting reflection on their embeddedness in institutions and perpetuation of intergenerational marginalization and injustices. Collins and Bilge (2020) have likened this phenomenon to ‘old wine in a new bottle’, highlighting how old injustices manifest in new technologies. In our analysis, the institution-focused approach is specifically directed towards the policies, practices, and dynamics within developers of vGenAI, such as Stability AI.

The third lens entails cultivating nuanced ethics that transcend single axes of oppression, instead addressing multiple power systems concurrently, such as capitalism, heterosexism, racism, and ableism (Matsuda 1990). It provides critical insights into how negative impacts such as stereotyping and exclusion could become exacerbated by this compounding, by exploring the interactions and co-productions between these systems. Social movements and critical social science theories provide valuable ideas for reflection and action for these purposes (Collins 2019). As explained by Sharma et al. (2023), for instance, decolonial work challenges the foundations of modern societies and knowledge by unsettling power relations, elevating submerged knowledge, and promoting ‘border thinking’ to center marginalized epistemologies (Dunford 2017). Anti-racist perspectives highlight reparative justice, interrogating White privilege and imperialist legacies (Benjamin 2019). Drawing from LGBTQIA + movements, queer theory disrupts sexual norms, questioning identity foundations and challenging heteronormative power structures (Gambino 2020). These diverse struggles uncover various mechanisms of injustice, from privatization to exploitation. This approach systematically engages these perspectives in dialogue, recognizing their commonalities, strengths, and insights.

## Methodology

As previously described, our intersectional inquiry into SDXL is underpinned by a methodological framework that combines both visual cultural, and intersectional perspectives, and it is deployed through an empirical analysis. This section outlines the methodology for collecting the empirical data for the analysis. When it came to the visual images, we used a specific prompting strategy to produce a dataset comprising 180 images using SDXL. This primary qualitative material was complemented by a review of online and academic sources that would help to understand the systemic embeddedness of the technology in focus. Furthermore, we engaged in a comprehensive review of critical literature to contextualize our qualitative findings.

### Research questions

Within the scope of this paper, we posed this overarching research question:

***RQ: How is Stable Diffusion producing privilege and discrimination in/through AI-mediated visual culture, from an intersectional perspective?***

Furthermore, we established three sub-questions that would contribute to answering the overarching research question. These sub-questions were derived from our analytical-theoretical framework that focuses on intersectional analysis on micro, meso, and macro-levels (see Sect. 2.4).

***SQ1: How are different social categories represented aesthetically in the imagery? (micro-level)***

***SQ2: How do different institutions come together to further marginalize certain social categories? (meso-level)***

***SQ3: How do various power systems intersect and come together as vehicles of oppression? (macro-level)***

### Data collection and analysis

**Fig. 2:**
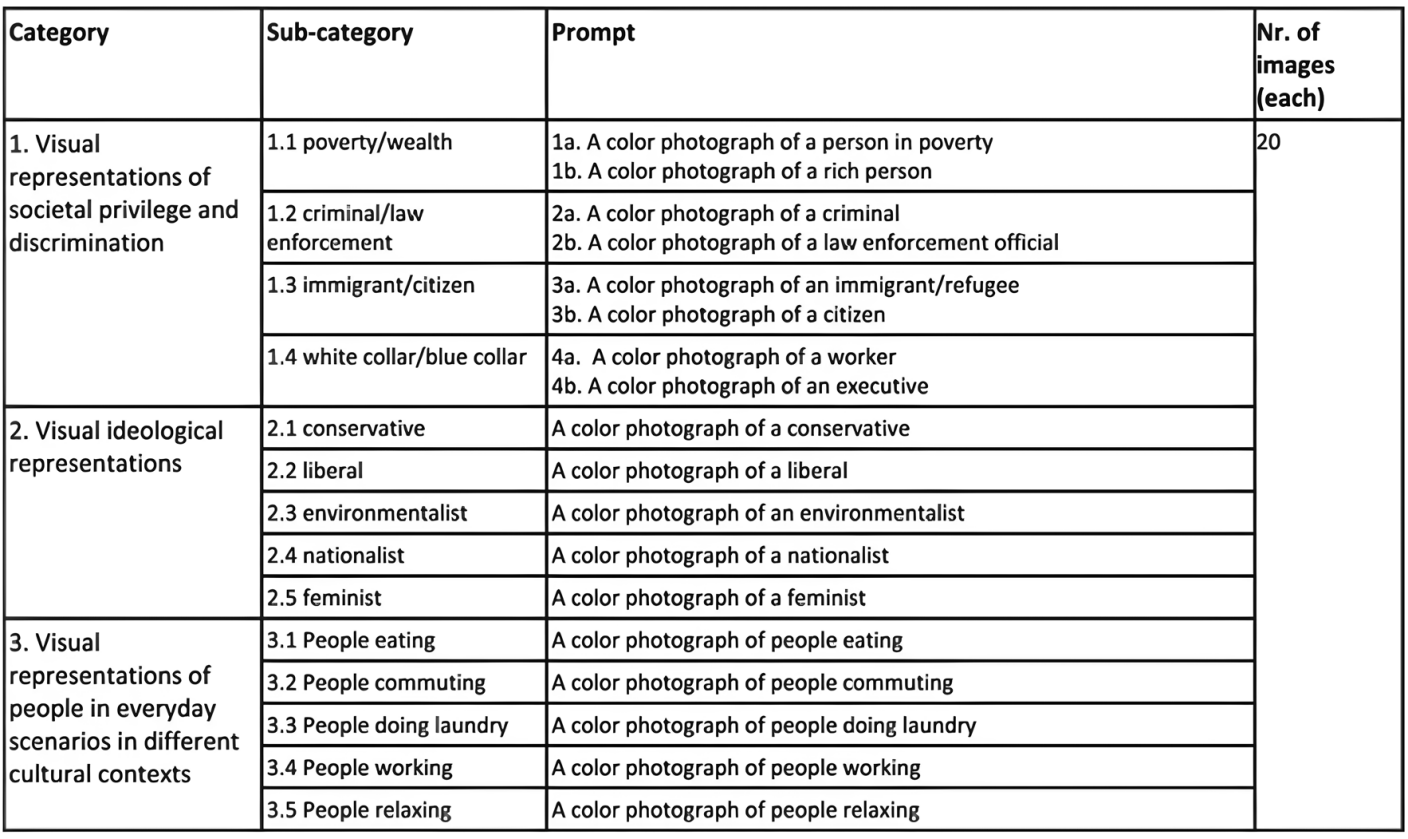
Qualitative data collection strategy for surfacing the AI imagery

At the outset of our analysis, the absence of a standardized methodology for prompting visual characteristics for intersectional analysis posed a challenge. Therefore, we initiated the data collection process by engaging in iterative experimentation with different prompts, aiming to capture the depiction of individuals, objects, and various cultural and everyday settings. This process helped us to establish three dimensions that we later on used in a more systematic and comprehensive prompting approach (see Fig. 2 for details). These dimensions included:

1. **Societal privilege and discrimination:** first, we analyzed divergent representational paradigms of societal privilege and disadvantage. These encompassed opposite prompts such as poverty versus wealth, criminality versus law enforcement, immigrant versus citizen, and blue-collar versus white-collar. The rationale was to facilitate an examination of visual depictions pertaining to privilege and discrimination in terms of resources and social status.
2. **Ideological representation:** second, we examined characteristics of ideological representation to uncover normative representations that relate social groups to ideology. These prompts included; conservative, liberal, environmentalist, nationalist, and feminist. These representations served to uncover the visual juxtapositions associated with the represented ideological stance and related social categories. We excluded other ideological representations, such as religion, for example, to limit the scope of the study.
3. **Everyday scenarios:** third, we decided to select a set of mundane scenarios which are shared in all human cultures regardless of the cultural background (e.g. eating, walking, working, washing laundry). The purpose of the last category was to uncover what visual features would be prevalent in these universal scenarios. This aids in uncovering the hegemonic visual cultural aesthetics by exploring how the imagery relates to different cultural contexts.

In our study, we intentionally used simple phrases in prompts when generating images. This approach was firstly motivated by the fact that it simulates the experience of everyday internet users who may lack specialized knowledge in prompt engineering. For these users, it is also hard to know what kind of model they are using and what the specific technical capabilities of those models are. Thus, we deployed a prompting approach that is not centering the system, but rather the users’ experience of using these unpredictable and non-transparent vGenAI tools. While in version SDXL 1.0 there might be an issue with representing West-African foods, in version 1.1 or 0.5 (as an example—these versions do not exist) these problems might not be present. This creates a situation, in which the users are unable to know the visual representations facilitated by the vGenAI they are using. This urges the need to develop systematic ways of analyzing the visual culture of these tools, such as we are doing in this study. While we recognize that more complex prompts—such as using additional text or negative prompts—can yield more nuanced outputs, our choice to use minimal prompts was deliberate. By keeping prompts minimal, we aimed to observe the default outputs and hegemonic aesthetics in the imagery. These are problematic from a visual cultural perspective because they create meaning by pairing the textual prompt and the aesthetic. For example, if people learn that a ‘man’ looks a certain way (for example, has short hair in every image), this predominant aesthetic can become normatively enforced in the visual culture. To give another example, if the everyday scenario of ‘eating breakfast’ is an American breakfast table, that similarly contributes to establishing the visual cultural norm for breakfast. For this reason, it is methodologically valuable to investigate the hegemonic representations facilitated by simple prompts.

In addition to this, we opted for the *‘photorealistic’* setting for all images, as it offers insights into how individuals are portrayed in highly realistic images that can potentially exert a greater influence on viewers. As briefly discussed in Sect. 2.1, the advent of the photographic image reshaped the role of images in modernity (Sturken and Cartwright 2017), a transformation that continued with the emergence of cinema in the 1890s, the evolution of electronic imaging in the 1940s, and, more recently, with digital imaging and AI-generated images. This photorealistic style (among other visual styles present in GenAI tools) highlights how each emerging visual medium builds upon and preserves the media that preceded it. It also holds the concept of photography as evidence for the truth—a claim which is also associated with AI-generated images.

As shown in Fig. 2, we generated 20 images for 18 prompts (360 in total), out of which 10 were randomly chosen (180 in total) for subsequent analysis. Our study employs an interpretative qualitative approach, focusing on examining the consistency of the visual aesthetics across the 180 generated images, rather than statistical significance. Thus, the 180 images analyzed are treated not merely as data points but as qualitative material that relates to the broader socio-technical landscape of vGenAI (Niederer and Colomb 2019), and visual culture. Qualitative researchers have argued that case study analyses are never neutral and are not observations from 'nowhere' (Flyvbjerg 2001; Alvesson and Sandberg 2022). It is therefore important to acknowledge positionality—identities and experiences—and how they influence the research process, both in the interest of transparency and to recognize the inherently political nature of knowledge (Moya 2011). To acknowledge this, we briefly disclose our researcher positionality. The first author, with a background in HCI, design, and visual arts, specializes in qualitative aesthetic analysis and critical approaches to vGenAI. The second author brings extensive experience in applying intersectional lenses to technology studies. The third and fourth authors, European experts in feminist humanities, enriched the analysis with insights from feminist cultural studies and STS. Our team’s diverse cultural, socio-economic, gender, and ethnic backgrounds led to specific choices in the research process and might have blinded us from some insights. For instance, we selected the food Fufu (Fig. 15) based on the experiences of our West African colleagues, and included comparisons between nurses (Fig. 14), inspired by our visits to India and observations of its distinct healthcare system. These choices, informed by our lived experiences, led us to examine certain areas where vGenAI may not fully capture the realities we have encountered. Our intent in sharing this account is not to detract from the rigor of our work, but to add transparency to our research approach.^3^

Supporting this, visual cultural analysis is always performed from three perspectives; there is the object, cultural context, and the viewer (Sturken and Cartwright 2017). Each of these three influences the interpretation of the image. Furthermore, qualitative visual analysis is often interpretative in nature and includes certain practices, such as documenting the imagery, writing descriptions of it, reflecting on it, and analyzing it (Gillian 2012). We have used this approach in analyzing qualitative imagery. In the process, we organized and presented the analysis according to three themes; *(1) visual representations of societal privilege and discrimination, (2) visual ideological representations,* and *(3) visual representations of people in everyday scenarios in different cultural contexts.* These themes were informed by intersectional theory on social categories, and provided the starting point for broadening our intersectional analysis on the micro-, meso-, and macro-levels (Sect. 2.2).

## Results

### Visual representations of societal privilege and discrimination

In this section, we begin by describing four different types of representations in images—poverty vs. wealth, criminality vs. law enforcement, immigrant vs. citizen, and blue collar vs. white collar. These four dimensions concern representations of social hierarchy, e.g. the visual markers in a socio-cultural system, in which power and status are assigned to social groups in different ways.  In what follows, we provide an overview of the analyzed images, accompanied with the qualitative analysis.

**Fig. 3:**
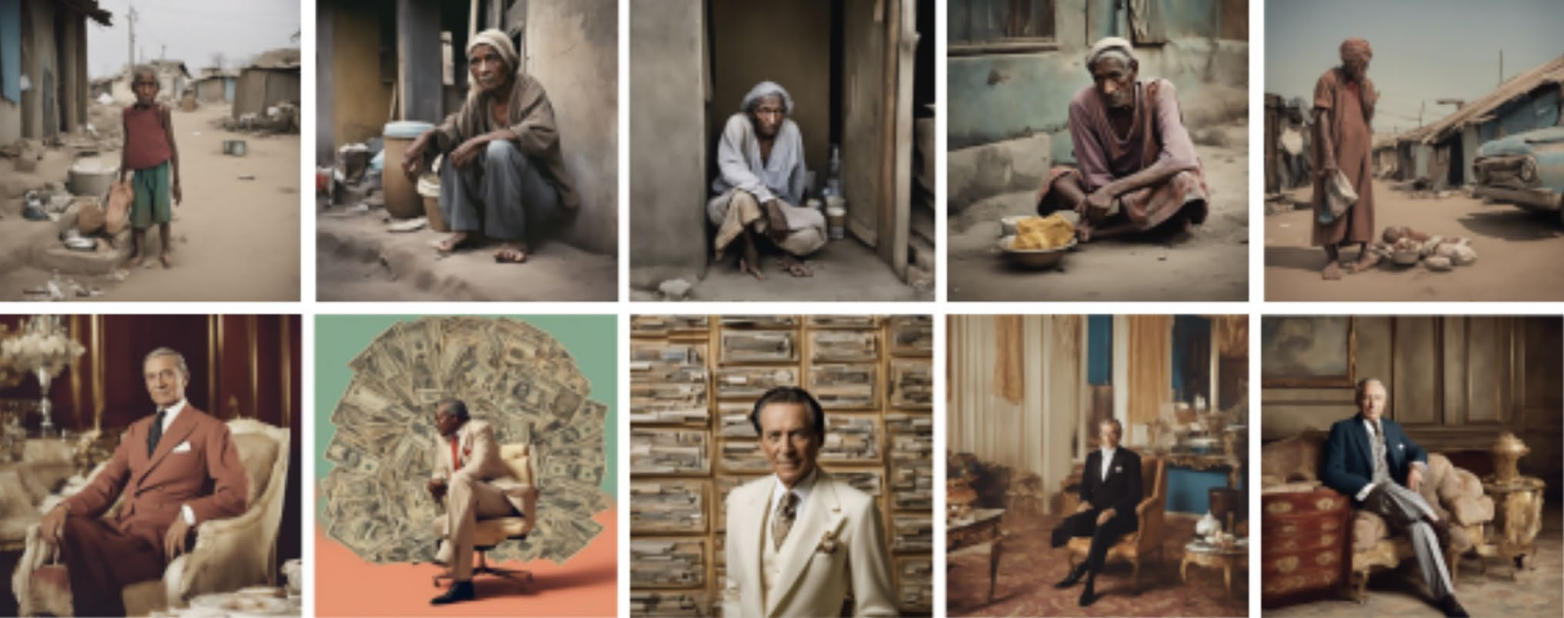
‘A color photograph of a person in poverty’ (above) v/s ‘A color photograph of a rich person’ (below)

In analyzing the aesthetics associated with ‘poverty’, it became apparent that representations of poverty exclusively depicted scenarios from the Global South, neglecting any portrayal from the Global North. This can be interpreted from the clothing of the people represented in the images—for example, the climate must be warm to wear sandals (Fig. 3), and the specific clothing styles featured on the ‘poor’ people, and the skin tones excluding any lighter tones. This raises questions about the mechanisms underlying this normativity and its potential association with processes of 'othering' (Southcott et al. 2020). In contrast, depictions of ‘wealth’ predominantly featured Caucasian masculine figures in settings reminiscent of the Global North (notable by the presence of dollar signs ($) in the background, and the furniture that resembles neoclassical style with its origin in Europe). No instances were found that clearly situated wealth in a different cultural context. Thus, a stark dichotomy emerges where the Global South is consistently portrayed as impoverished, and masculine figures of Global North as wealthy.

**Fig. 4:**
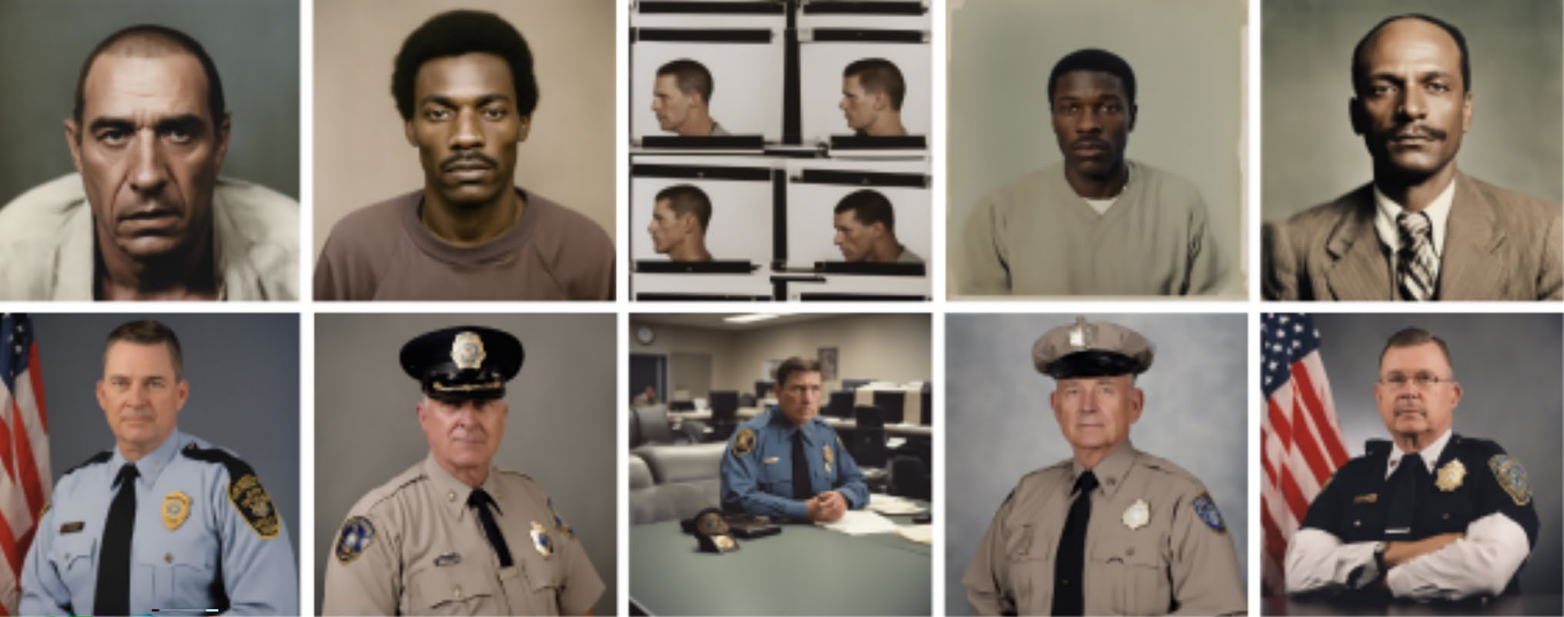
‘A color photograph of a criminal’ (above) v/s ‘law enforcement official’ (below)

**Fig. 5:**
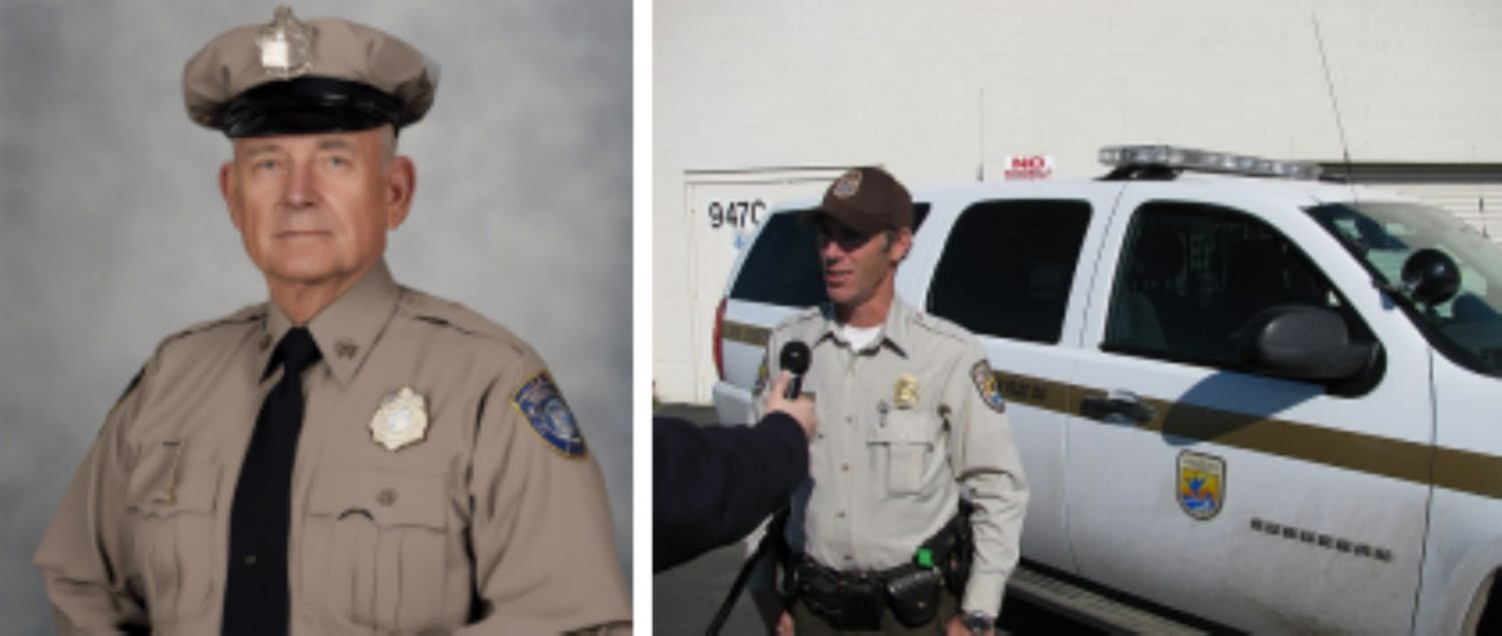
Comparison of the outfit of an 'American police' (Pacific Southwest Region 2011) and the police in the AI imagery

Advancing to the portrayals of criminality and law enforcement (Fig. 4), law enforcement was commonly portrayed with United States (US) flags on the background and exclusively caucasian masculine faces. The police uniforms also resemble uniforms used in the US (see comparison in Fig. 5). For criminality, the imagery resembled mugshots and portrayed a wider ethnic diversity. This gives an impression that law enforcement is ‘white’, but criminality is more diverse in terms of social groups. This imagery likely reflects on the cultural setting of the US, which has recently been under critical discussion for the systemic racism that is also deeply integrated in the law enforcement system (e.g. black lives matter)—and apparently even the AI imagery that depicts law enforcement. It is also essential to acknowledge that both criminal and law enforcement depictions lacked feminine representations, thereby excluding other gender representations than the masculine heteronormative norm from the portrayal of law enforcement.

**Fig. 6:**
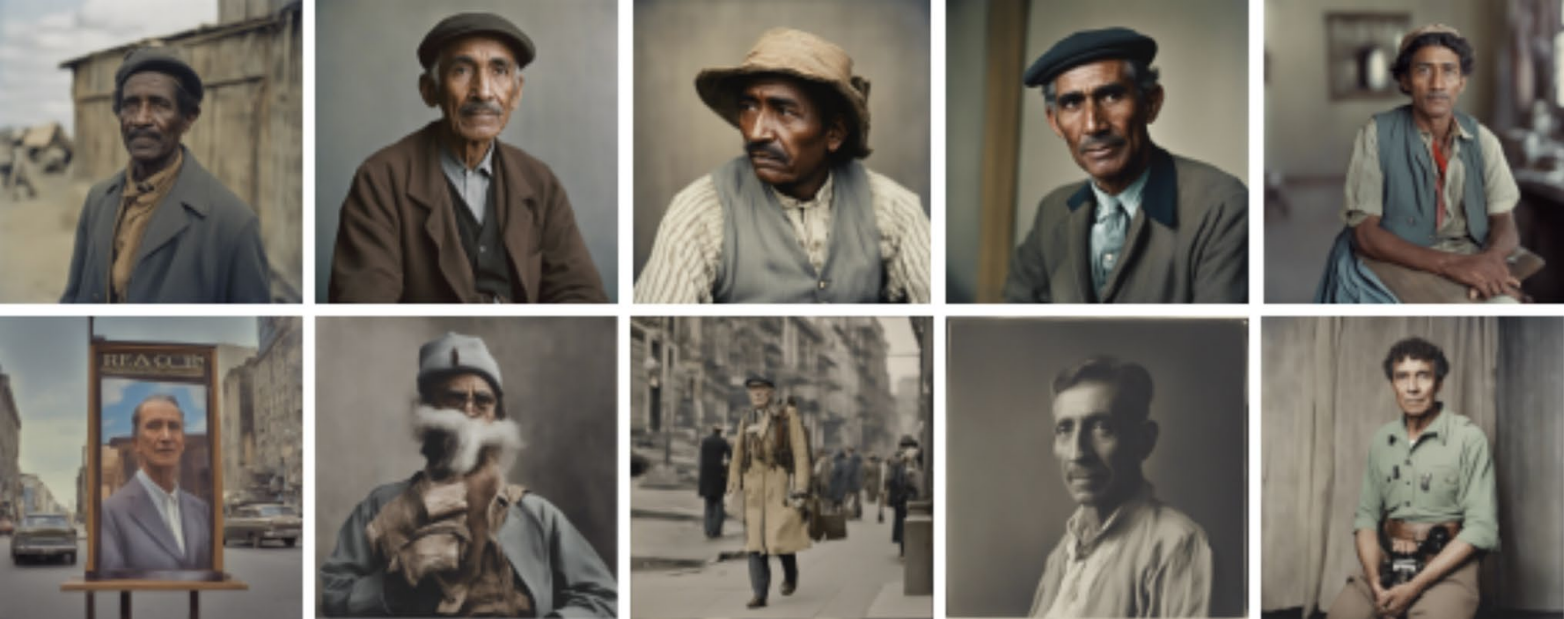
‘A color photograph of an immigrant/refugee’ (above) v/s ‘a citizen’ (below)

**Fig. 7:**
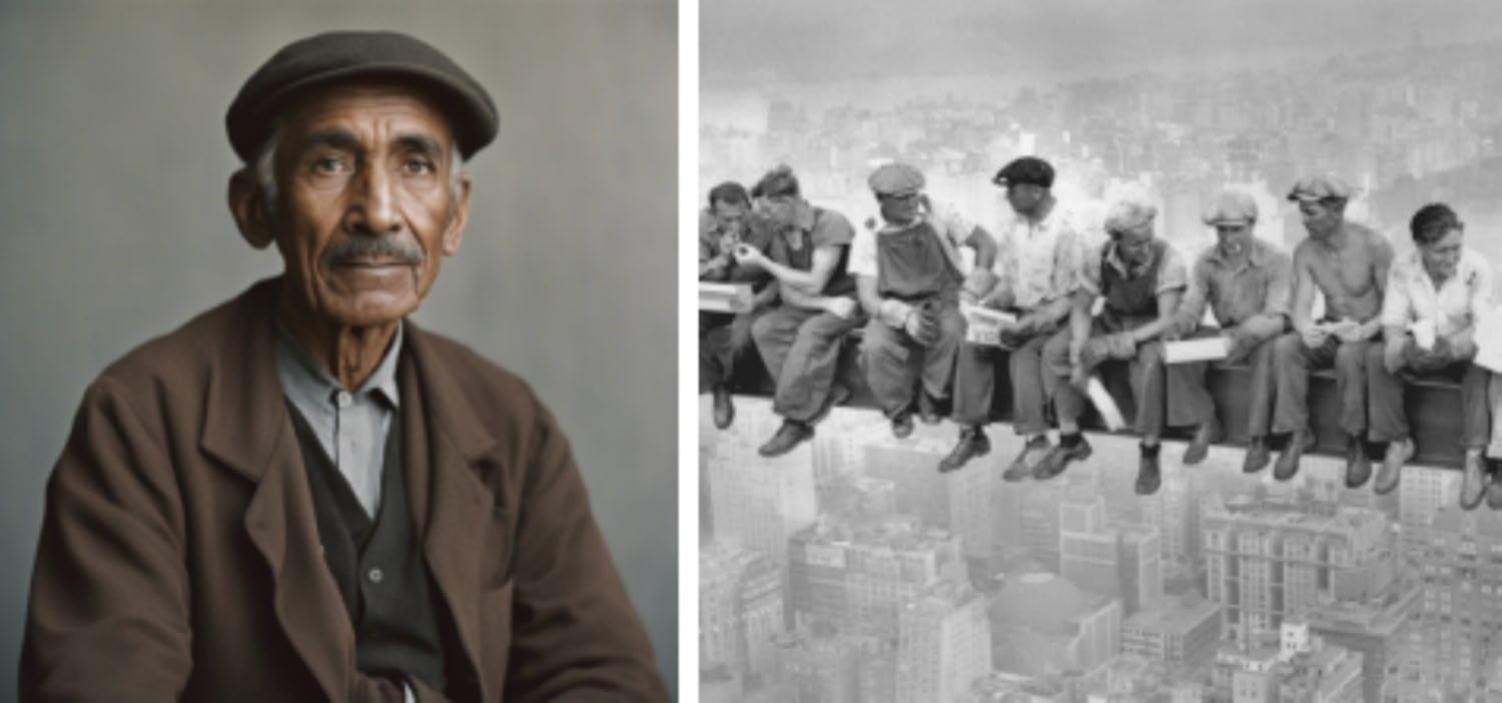
Outfit from 1800s resembling those of the AI imagery (Acme Newspapers 1932)

The portrayals of the immigrants and citizens also featured primarily masculine representations, excluding feminine and non-binary representations (Fig. 6). The immigrants, again, featured only darker skin tones, whereas the citizens had a wider variety of representation. The clothing in these images seemed to eerily belong to a certain era of 1800–1900s (see similar outfit in Fig. 7 from 1900s America worn by blue collar workers). Otherwise it was challenging to discern the historical context of the aesthetics in this imagery category.

**Fig. 8:**
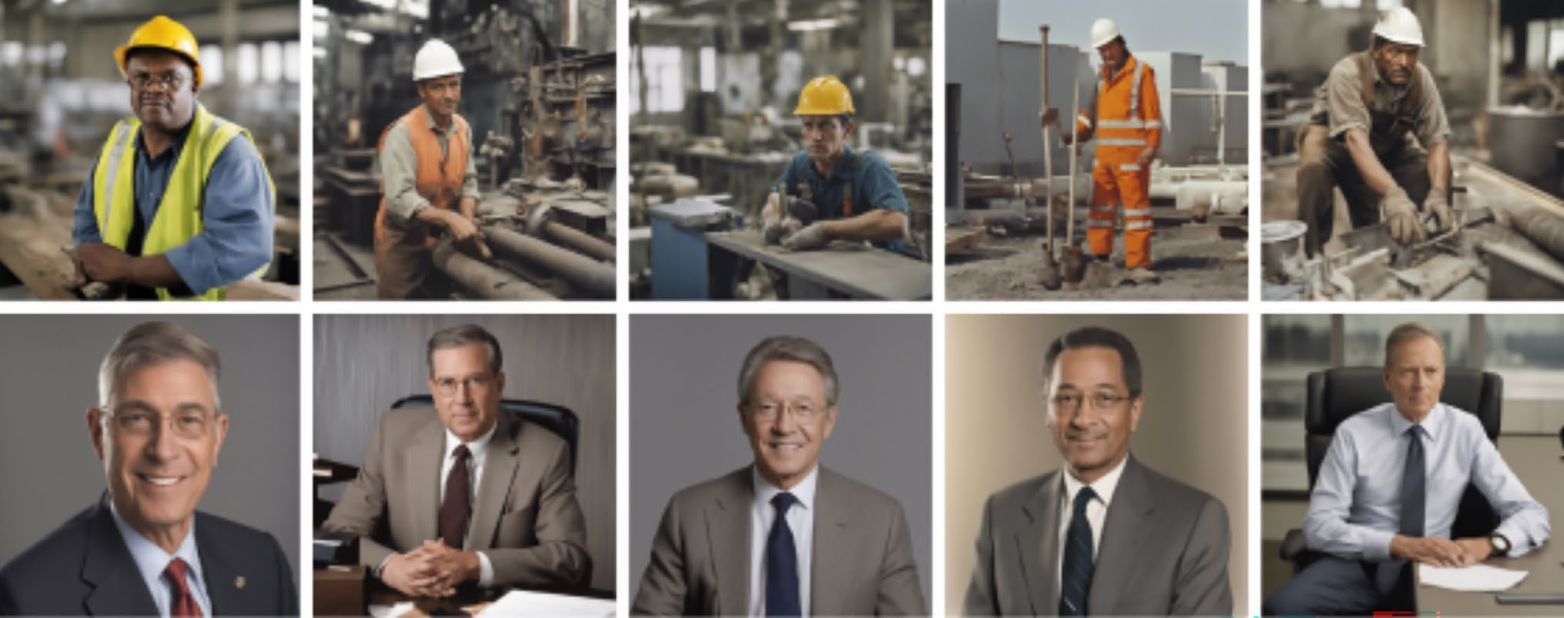
‘A color photograph of a worker’ (above) v/s ‘an executive’ (below)

**Fig. 9:**
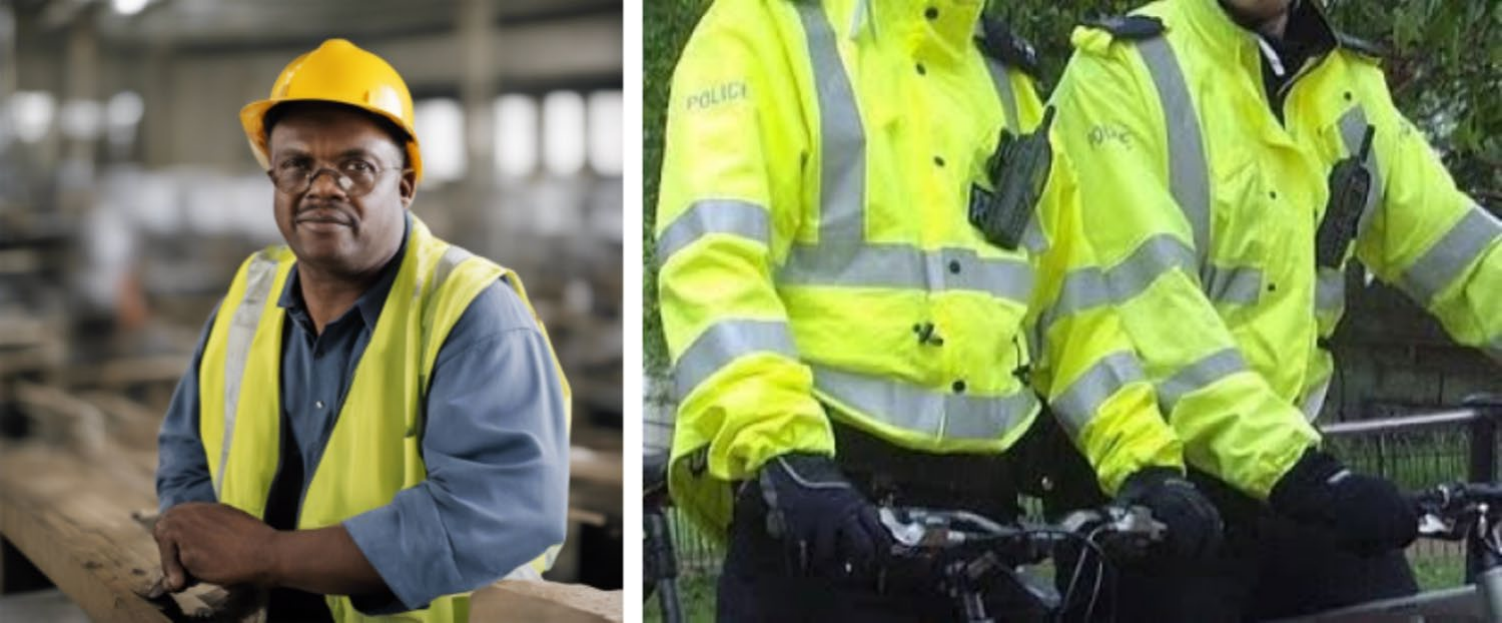
High visibility vests that indicate the imagery is taken after the 1930s – right image from web (Furtado, 2010), left image generated with SD

In terms of representation of labor classes (Fig. 8), the imagery also lacked diverse gender representations, and there was a clear pattern visible in the clothing styles of the blue collar and the white collar. The blue collar featured safety hats used in construction sites in all examined images, and safety vests and clothing in milieu that represented construction sites. The imagery of white collars featured ties and suits. The contextual analysis allowed us to situate these images within specific historical eras, discernible through depicted items. For example, high-visibility fabric paint was invented by Bob Switzer, who in the 1930s was injured in a workplace accident (Kane 2014). These high visibility vests that require the fabric paint were thus common from the 30s onward, situating the imagery in the following decades (see Fig. 9). On the other hand, the suit garment seen in the white collar images has also existed in earlier decades, but the color of brownish and gray colors was particularly popular during the 1970–90s.

When examining the gender portrayal in all of the imagery above, it was evident that the aesthetics predominantly featured adults conforming to traditional heteronormative gender aesthetics. Across various images, gender assignments were automatically applied by SDXL, resulting in aesthetics that excluded feminine or non-binary representations. This absence was particularly notable in categories of criminality, law enforcement, citizenship, and immigration, where stereotypes of masculine figures were consistently depicted. This lack of diverse gender representation aligns with many other studies in the domain (Almeida et al. 2024; Jääskeläinen et al. 2024; Offert et al. 2022; Anand et al. 2023; Luccioni et al. 2023) and raises concerns about inclusivity for gender minorities by perpetuating normative visual culture exclusive to certain gender identities.

When it came to ethnicity, we observed quickly that regardless of the prompted domain, the representations were often as a default focused on ethnically caucasian-looking representations of people. An exception to this was the material presented above, in which the privilege (such as wealth or citizenship status) was attributed to the white skin color, and the disadvantage (such as criminality) was connected with darker skin tones. This creates an absurd visual juxtaposition, which yet is very concrete and rooted in the real society and its systemic discrimination practices. When we further prompted specifically for a certain ethnicity outside of the predominant norm, the change took place superficially – for example, by only switching the skin tone and not the cultural settings/background. We will discuss this further in Sect. 4.3.

The imagery also displayed specific age aesthetics. While the majority of the images depicted middle-aged adults, certain categories, such as environmentalists (see analysis in Sect. 4.3), featured different age representations. These age representations exhibited ageist tendencies by largely excluding both older individuals and younger people, including children. This pattern was consistent across all prompted categories, with the exception of poverty. Regarding physicality, the analyzed imagery predominantly featured adults with typical body sizes, types, and abilities. Notably, there were no representations of disabilities, such as prosthetics, and a notable absence of individuals with larger or smaller body sizes. Consequently, unless specifically prompted for diversity, the AI-generated imagery lacks diversity to a significant extent, perpetuating ableist norms by portraying stereotypical bodies with uniform characteristics as the visual standard, based on what we witnessed with the 180 samples gathered for this study.

### Visual ideological representations

**Fig. 10:**
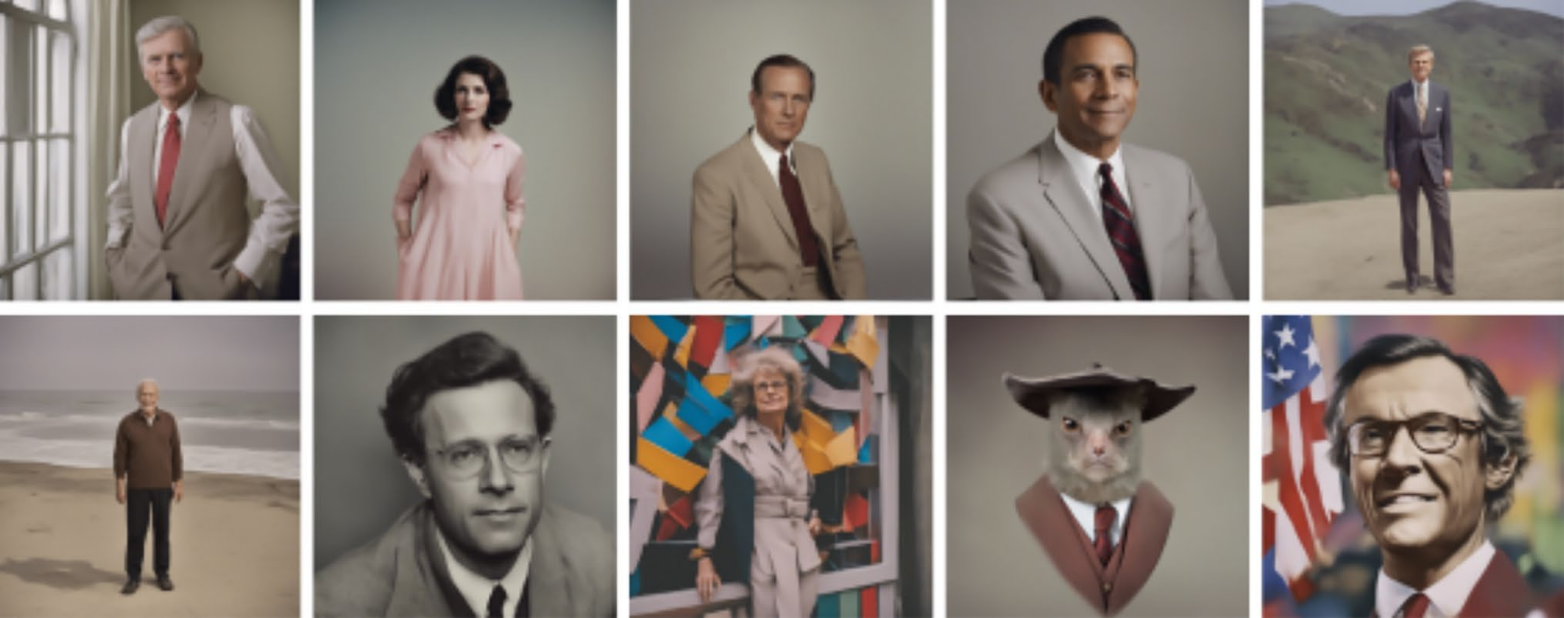
‘A color photograph of a conservative’ (above) v/s ‘liberal’ (below)

When prompting for ideology, we observed how certain social groups became associated with certain ideologies. ‘Conservative’ figures were often depicted in outfits reminiscent of 1950s gender stereotypes, particularly featuring caucasian figures from the US (Fig. 10). Heteronormative masculine attire resembled that of white-collar professionals, with suits being a common motif. Liberal representations typically included US flags and exhibited greater diversity, although predominantly featuring masculine figures. Some images featured random elements, such as a rat face, alongside numerous individuals wearing glasses, possibly representing academics or intellectuals. Notably, all representative figures were Caucasian, and the imagery bore stylistic resemblances to the 1970s–1990s, with colorful backdrops and artistic elements suggesting a connection to libertarianism and artistic expression.

**Fig. 11:**
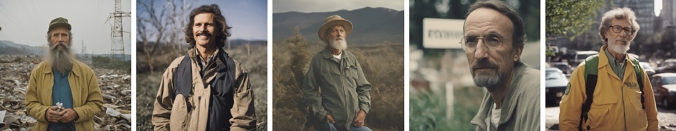
‘A color photograph of an environmentalist’

While prompting environmentalists (Fig. 11), images depicted older caucasian masculine representations in outdoor settings, such as hiking. Given the contemporary diversity of environmental advocates, exemplified by figures like Greta Thunberg, these depictions appeared outdated, reminiscent of environmental activists from the 1970s-90s in the Global North. It's noteworthy that significant environmental advocacy existed as early as the 1960s, challenging the dominance of heteronormative masculinity in scientific circles (Carson 1962). Many depicted environmentalists also appeared elderly, characterized by white hair and glasses, suggesting an older demographic compared to the previously described imagery.

**Fig. 12:**
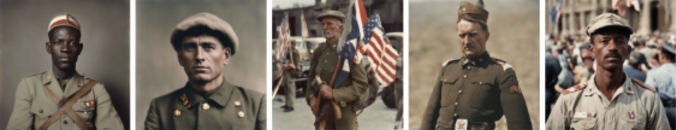
‘A color photograph of a nationalist’

When prompting for a ‘nationalist’, we observed ambiguous connotations to a confederate flag and the US national flag (Fig. 12). The images evoked connections to civil wars, notably in the US and possibly Africa, as suggested by the first image. However, due to their randomness and lack of clarity, drawing definitive conclusions proved challenging. The imagery neglected more current representations of nationalism—which might have been very well censored from the training data or imagery (e.g. Google’s Gemini placing people of colour in Nazi-era imagery, Al Jazeera 2024). Like other prompted images, these depictions were situated within specific historical contexts rather than being contemporary. The images seemed to perpetuate an 'old visual culture', potentially due to reliance on outdated training images. Thus, SDXL lacks explicit awareness of temporal and geographical factors, which could lead to problematic generalizations. It seems to switch between various eras and cultures and their visual characteristics and norms, without making this explicit to the user in any way.

**Fig. 13:**
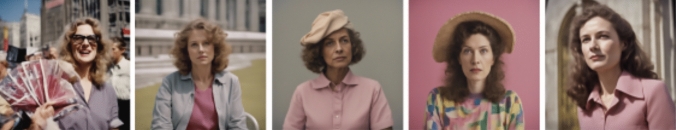
‘A color photograph of a feminist’

Lastly, prompting for ‘feminist’ produced exclusively feminine representations (Fig. 13), and featured predominantly pastel colors in the clothes and backdrops, located in the 1960s-80 s. Many of these images also featured caucasian feminists, portraying them as potentially well-educated and empowered. This interpretation was grounded on how the backdrops of the images seem to feature university buildings or other prestigious institutions with architecture from the Global North. This imagery is also clearly outdated, lacking the diversity of ethnicities, gender, abilities, and geographies of contemporary feminist movements. It is also notable how most clothing features pink color, which became associated with feminine aesthetics after a deliberate cultural campaign around the 1900s (Grannan 2024).

### Visual representations of people in everyday scenarios in different cultural contexts

We argue that the history and heritage of the generated images should be highlighted, rather than obscured. It is critical to consider the imaging conventions behind who is represented and how, as the training data for AI models reflects the perspectives of the image and technology makers. Our investigation revealed a significant influence of Global North hegemony in the imagery, with much of it depicting US settings. Moreover, many images spanned the 1800s–1900s, particularly in categories like immigration and citizenship. This temporal focus could reflect the limits of the training images, constrained by copyright expiration and the rise of modern photography.

**Fig. 14:**
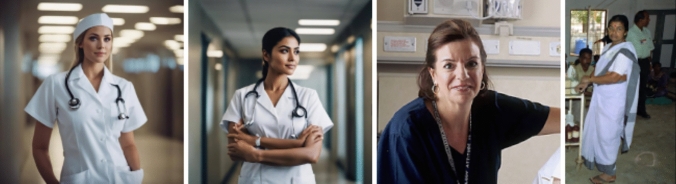
Prompting for ‘nurse’ and ‘Indian nurse’ vs. real photographs of nurses in the USA and India hospitals (Baer 2007; Government of India 2004)

Through further experimentation with the tool, it became evident that altering the contextual prompts for images only led to superficial changes while preserving an entrenched cultural aesthetic context. For instance, during our initial exploratory phase, we observed that requesting images of working-class individuals, such as nurses, typically yielded imagery reflecting a hegemonic portrayal prevalent in the Global North—a depiction of a white nurse in a pristine hospital setting. However, modifying the prompt to specify an ‘Indian’ nurse or another alternative cultural context resulted in minimal changes to the background of the images, with only the facial features of the individual changing (Fig. 14). Consequently, despite the altered prompt, the underlying aesthetic hegemony persisted, underscoring the superficial nature of the image alterations. This phenomenon arguably intersects with both cultural colonization and cultural appropriation: cultural colonization is evident in how the imagery is molded to conform to the aesthetics of the dominant culture, while cultural appropriation is manifested in the reductionist portrayal of the cultural context.

In many ways, performing this type of analysis of the image aesthetics resembles ‘AI archeology’ that involves examining the scrambled traces of locations and times through the involved objects and representations. It is therefore essential to involve people who have skill sets in reading historical images in this type of image analysis (historians, art historians, etc.), as also noted by (Jääskeläinen et al. 2024). Some relevant examples that could inform such studies include Forsythe (2001) and Mackenzie (2017). The former is an anthropological study of machine learning system development practices, whereas the latter is an archeology study of historical machine learning data practices.

**Fig. 15:**
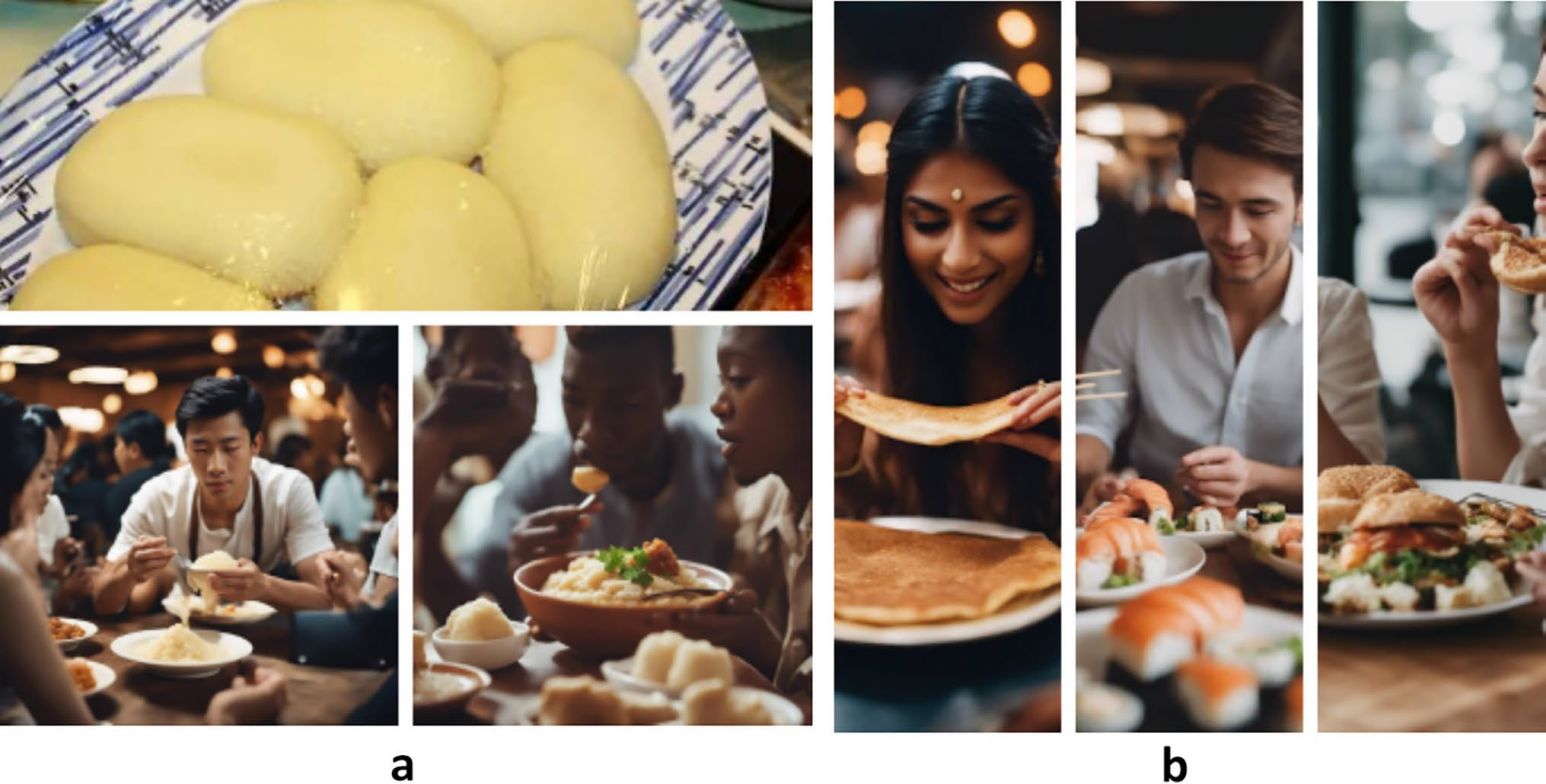
**a** An image of the dish fufu (DaSupremo 2020) in contrast to prompting 'people eating West-African dish of fufu', which looks more like mashed potatoes. **b** An image of 'people eating dosa/sushi/burger' – SD seems to be more accurate when it comes to more mainstream foods from other cultures

We also extend the prompting to general, less value-laden scenarios depicting everyday life, as these prompting strategies prove useful in understanding what is centered in the visual representations of the model. We observed similar visualization patterns to what we described earlier while prompting for everyday activities—the predominant representation for the general prompt of ‘people eating’ involved, once again, caucasian-looking people eating stereotypical foods, such as pizzas. Then, changing the prompt into these people eating something else (e.g. dishes from minority cultures), the change would take place superficially in the color or texture of the food, but similar aesthetics for the meal setting would prevail (e.g. middle class or well off people eating in a restaurant—see an example in Fig. 15). We are not showcasing all the results from this stage in the paper, but rather highlight the most interesting insights. During our exploration phase, we prompted SD to produce images of people eating the West African food Fufu (or FouFou). Based on the results, Fufu was first misunderstood as a Chinese term, and the results generated were mostly of people with East Asian features, eating dishes that possibly look like mashed potatoes or rice. When we prompted more specifically with ‘African Dish’ at the end of the prompt ('People Eating Fufu African Dish’), it resulted in something that looked closer to Fufu, and consisted of people with darker skin, and African features. However, the food itself still resembles something like mashed potatoes, rather than accurately depicting the dish. In the other example (Fig. 15b), we prompted some well-known foods from other cultures, such as dosa, sushi, and burgers. SDXL was able to depict these foods slightly more accurately, although the same polished style of middle class figures is present in the images. The dosa also looks slightly thick for a food that is usually quite thin—which creates more of a resemblance to pancakes. Thus, our results illustrate traces of both digital cultural colonization wherein imagery is transformed to align with hegemonic cultural aesthetics, and cultural appropriation where imagery fails to capture the complexity of cultural contexts and instead presents a reductionist portrayal.

## Intersectional analysis

In this section, we draw from and build upon the empirical interpretative image analysis by deploying the three-level intersectional framework. This analysis is structured following our three research sub-research questions.

### Micro level—how are different social categories represented aesthetically in the imagery?

As evidenced in the visual analysis above, a discernible pattern emerges wherein a specific social category predominates a particular subset of images and aesthetics. Notably, wealth is exclusively associated with heteronormative masculine depictions, whereas feminism is exclusively associated with heteronormative feminine portrayals. It is imperative from an intersectional standpoint to highlight that not only are wealth and feminism portrayed as inherently masculine and feminine, respectively, but they are also depicted through a narrow lens encompassing only certain manifestations of masculinity and femininity—white, cisgender, able-bodied adults, and conventionally attractive. Thus, SD inadvertently excludes specific representations of individuals, such as wealthy people of color, economically disadvantaged white individuals, transmasculine/cis-masculine feminists, and notably, feminists of color across all genders. For instance, the absence of feminine figures or people of color in response to a prompt for an environmentalist, particularly in an era marked by public figures such as Wangari Maathai, Greta Thunberg, and Disha Ravi, underscores the skewed representations produced by SD.

Perhaps most fundamentally, what our intersectional approach reveals—which would not necessarily be so apparent if we had chosen to focus on, for example, gender or ethnicity alone—is that in many cases the hegemonic representation that is constructed through the SD imagery is a figure who is white, adult, able-bodied, and heteronormative masculine presenting. Where the model appears to be attempting to show a greater diversity of individuals in its imagery, it tends to change just one characteristic from this norm, resulting, for instance, in one man of color appearing in the images of wealth, or one white woman appearing among the ‘conservative’ images. This mirrors and perpetuates the dynamics which have been described by Crenshaw, Collins, and many other intersectionality theorists over the years, that diversity of representation is often only imagined across one axis of differences, and the complex intersections of these axes are rarely explored. Additionally, given the significance of visual media representations of professions in career decisions, the impact of perceived inadequacy in matching dominant professional images on individuals could have a significant impact on individuals’ career choices, further aggravating inequalities. This internalization of knowledge about the world can solidify certain truths that are challenging to unlearn. For example, academic research has underscored the discouragement faced by women, particularly women of color, in pursuing certain career paths due to the lack of diverse representation in visual culture (Gorska & Jemielniak 2023). Furthermore, as AI-generated images explode and flood the internet, there are already epistemic challenges in distinguishing AI-generated images from human-generated ones (Jiang et al. 2023).

### Meso-level—how do different institutions come together to further marginalize certain social categories?

Institutions and their histories have a central role and influence on social justice (Williams 2021). This also applies to vGenAI technologies—such as SD—which largely originate from the Global North. SD emerged from the collaboration between Runway, a US-based AI video generator, and researchers at Ludwig Maximilian University in Munich, with computational support from Stability AI, and is trained on the LAION-5B database (Rombach et al. 2022). The BigTech landscape in the US is known for dismissing concerns of gender, race, class, and sexual identity, obstructing efforts aimed at breaking down discriminatory barriers and hindering systemic change (Tacheva and Ramasubramanian 2023). A good example is the industry-based research published by Bender et al. (2021) that highlighted the potential discrimination of AI models and consequently led to the firing of one of the authors from Google’s responsible AI team (Guardian 2023). Furthermore, this BigTech culture in which Western vGenAI companies are embedded is also known for ethics-washing, and superficially aligning with social justice concerns while discouraging efforts for deeply reflective and critical practices in reality. Furthermore, Benjamin (2019) highlights how even in attempts to become more inclusive toward various groups of people, it is often done in exploitative ways. Such superficial diversity initiatives fail to address systemic issues, such as algorithmic injustice, and indicate a detachment from the history that underpins discriminatory (racist, sexist, ableist, etc.) practices in technology development (Small 2023).

Contextualizing this general picture with specific examples and criticisms of SD, Stability AI’s founder, Emad Mostaque for example has been “*sensationally accused of exaggerating his education, the genesis of AI image generator Stable Diffusion, and links to prestige organizations such as the United Nations”* (Growcoot 2023), using similar ethically questionable strategies demonstrated by the AI companies generally. It has also been claimed that Mostaque has obscured the credits of creating SD to the German research group which created it, quickly making a press release and positioning himself as its chief evangelist. Despite these misconducts, Stability AI managed to secure $101 million USD in funding in 2022. This shows a critical problem in how the capital and market steer the technology forward—in the absence of ethical vetting or checks that would prioritize examinations of the technology, its imagery, or the practices of the company. It is also notable that AI technologies often gravitate towards free markets—such as the US—to avoid regulation and accountability (McElroy 2024). It is notable that governmental bodies such as the EU have recently significantly emphasized AI regulation, putting forth for example the new AI act (European Commission, 2023), in an attempt to gain some control over this. Stability AI has also hidden behind not-for-profits such as LAION-5B (which are in turn partly funded by BigTech) to avoid legal scrutiny since databases created for research purposes are given more leniency in courts when it comes to copyright issues (Jiang et al. 2023). SD is a good example of how research becomes privatized and misappropriated for the profit-making intentions of BigTech since research institutes do not have access to the same level of funding. To extend the list of currently reported misconducts in the case of SD, they were also sued by Getty Images for stealing photos for the model training (Korn 2023) and Stanford researchers found that SD was trained with a 1000 images of child sexual abuse (David 2023). Thus, SD has a prior history of overlooking regulation and seems like a textbook example of various unethical practices in the design of vGenAI technology. We can also clearly observe various institutions and systems—such as the tech industry and its practices, education of technologists, regulations set by the government—to come together as an institutional context in which the systemic injustices reside and get reproduced. As our analysis demonstrates, this is observed both in the visual culture and through various evidence of societal practices and organization.

Scholars emphasize the inadequacy of policy-led regulation alone in holding the tech industry accountable, advocating for a shift in machine learning researchers' *perspectives* toward recognizing the intersection of technology with power dynamics (Tacheva and Ramasubramanian 2023). Addressing the neocolonial and capitalist tendencies of vGenAI, while promoting societal justice-oriented cultures within vGenAI organizations is crucial for fostering equity. This transformation should address the specific systems of oppression on multiple levels—the technology and the socio-technical structures around it, while emphasizing the power systems that position various social groups in ways that configure privilege and disadvantage. In practice, this could mean working towards reconfiguring the aesthetic representations of social groups, while also aiming for societal systemic transformation through regulation, civil action, and other means for social change.

### Macro-level—how do various power systems intersect and come together as vehicles of oppression?

In the final analysis, we deal with power systems, exploring how systems of oppression such as racism, capitalism, and colonialism intersect particularly in the case of SD. Power systems are essentially beliefs, cultural norms, and practices (Guillaumin 2002) that reinforce certain ideologies, such as heteronormativity, patriarchy, racism, and ableism. As discussed in the earlier part of the analysis, we can clearly observe how various power systems, such as racism, sexism, capitalism, and digital neocolonialism are intertwined and present in the case of SD, and pierce through both the visual culture and the institutional context. More concretely, through our analysis of the imagery and institutional context of SD, we were able to demonstrate that SD is embedded in and perpetuates power systems that support these specific ideologies; racism, sexism, hetero-normativity, ableism, ageism, and capitalism. We also saw how aesthetics and data embedded in a certain cultural context lead to reproduction of the power systems in that cultural context. While it is nothing new that 'biases' from society get embedded into the technology, we demonstrated  in the case of the specific technology of SD how its aesthetic and visual cultural outcomes work as a vehicle of oppression from an intersectional perspective.

Our results regarding cultural representation (see Sect. 4.3, Fig. 15a, b) illustrated both digital cultural colonization in which the imagery is transformed to align with hegemonic cultural aesthetics, as well as cultural appropriation in which the imagery fails to capture the complexity of cultural contexts and instead presents reductionist portrayals. Based on our analysis, we argue that SD derives advantages from the hegemonic power structures. We advocate for the urgent need for forthright acknowledgment of these power structures (e.g. acknowledging the visual politics of portrayal of vGenAI technology, such as SD), along with their historical and contemporary ramifications on marginalized communities, thereby facilitating their redressal and fostering a culture of accountability under the purview of restorative justice. Decolonial perspectives have shed light on persistent power imbalances inherited from colonialism, which influence contemporary knowledge production and labor relations (Mohamed et al. 2020). The enduring effects of historical appropriation, dispossession, exploitation, and control structures established during colonization underscore the need to critically examine the role of colonial ideologies in shaping vGenAI technologies (Arora et al. 2023). In our image analysis, we observe a clear manifestation of the imposition of aesthetics and cultural norms of the Global North embedded in AI systems. When these norms and aesthetics are skewed towards the centers of power of AI (Jiang et al. 2023), they work as vehicles for both cultural colonization and cultural appropriation.

As discussed in Sect. 5.2, vGenAI technologies have garnered substantial investment from venture capitalists and are disrupting traditional industries, potentially reshaping entire economies and social structures (Verdegem 2024). This competitive landscape could exacerbate economic disparities and consolidate power among BigTech firms. This, in turn, can lead to increased perpetuation and maintaining systems of oppression—which are already embedded into the ‘AI Empire’ (Tacheva and Ramasubramanian 2023). As exemplified in our analysis in Sect. 4.2. (ideology and social categories) in Fig. 12 which resembles a nationalist soldier holding a confederate flag, our results seem to generate ambiguous images which might propagate violent (e.g. colonial) symbols—such as the confederate flag. In our analysis of the generated images, we identified the confederate flag as one of the most explicit violent symbols that appeared. We described it as violent due to its strong association with slavery and systemic racism, which continues to impact many communities today. This symbolism is especially significant in the context of the Black Lives Matter movement, where such imagery has been widely condemned for perpetuating harm and reinforcing racial oppression. Racist iconography and dehumanization of Black people continue to exist in SD (Keenan 2023), and its content has been misappropriated by far-right elements to create politically charged images that fit their narratives (Bitter 2024). Thus, racist algorithmic outputs find new ways of marginalizing Black people while being shielded by the capitalist, commodification-driven intentions of their makers. Furthermore, as these technologies rely on vast amounts of computing resources, predominantly controlled by major tech corporations, concerns arise regarding commodification and extraction, further entrenching power imbalances (Weidinger et al. 2022). Several formerly colonized nations are rich in these resources, and developing nations such as those in South Asia provide cheap labor which is exploited by AI technologies to train algorithms or label data (Bitter 2024). Thus, it should be highlighted how these postcolonial arrangements of injustice are deeply entangled with their capitalist modes of operation, and how the various power systems that we discussed above are deeply intertwined with each other.

## Discussion and conclusions

In this paper, we have uncovered power configurations that embed particular dynamics of privilege and discrimination in the images produced using SD, and which have the potential to significantly shape the global visual cultural landscape. We began with the empirical image analysis, which surfaced discrimination toward various minority cultures. The imagery of SD most often strengthened binary gender representations while prioritizing white masculine representations at the expense of other social groups. Furthermore, non-binary gender representations were absent due to the strong binary gender aesthetics, lighter skin tones were associated with wealth and societal status, whereas darker skin tones and cultural contexts outside of the Global North were often associated with poverty, criminality, and other negative social judgments. We also discovered a strong tendency towards ableist imagery and witnessed a lack of variety in age representations with young adults as the most common representative group. When it comes to the social and cultural context of the technology, we demonstrated that SD is deeply embedded in and emerging from capitalist profit-seeking motivations that tend to overlook social justice and that the problematic imagery is symptomatic of the problematic visual culture and power structures at large in the societies that give birth to these technologies. By examining the visual aesthetics of vGenAI, we uncover the complex systemic power structures—and therefore highlight that it is not enough to address the issues superficially in the imagery alone. Instead, a more comprehensive and radical systemic change needs to take place in society at large. If the underlying societal power structures are not reformed, it is inevitable that similar neocolonial technologies keep getting produced to work as vehicles of oppression toward certain social groups. This speaks to the importance of finding ways for radical socio-political approaches to re-imagine and re-configure the society and its AI-aesthetic landscape.

To further highlight these concerns, superficial fixes of the vGenAI aesthetics can lead to 'diversity washing', in which the underlying societal problems are not fixed—and the companies providing the technology claim it to be 'diverse' and 'neutral', without fully understanding the aesthetic cultural impact and politics of the very technology they provide for large-scale use. We discussed earlier how the vGenAI images are embedded within and constitutive of broader power systems of racism, capitalism, through the concrete example of the confederate flag. Paradoxically, while symbols such as the confederate flag are present, at the same time some other colonial and oppressive symbolism is censored—such as the symbols used by the Nazis. This showcases a superficial fix to address the problematic visual aesthetics without understanding the visual cultural embeddedness of vGenAI tools, and value-laden cherry-picking which and whose oppression is rendered visible or invisible in the visual culture.

In our generated SD imagery, we observed several visual 'glitches', such as the inability to accurately depict cultural items like fufu and remixing aesthetics from different cultural contexts in absurd ways. These limitations suggest that vGenAI software simplifies the complexity of the real world into reductive representations—an effect that some might dismiss as mere visual glitches due to the nature of machine learning processes. Building on Benjamin’s argument (2019), these visual glitches reveal more than just technical limitations; they expose the underlying power dynamics in algorithmic design. Inconsistencies we discuss in our analysis may often be dismissed as simple algorithmic ‘dumbness’ (Lieber et al. 2024) or attributed to issues of representation within the training data. However, they reflect systemic power imbalances embedded within vGenAI technology, leading these systems to perpetuate racial, gendered, and class-based inequities. Seen through this lens, the glitches in our generated images are not neutral mistakes but indicators of how intersectional power imbalances shape algorithmic outputs. We also discussed, how the imagery continually others and excludes non-Western cultures, by, for example, misrecognizing and representing foods and cultural settings. This actively contributes to neocolonialism through visual culture, similar to how Hollywood cinema has been criticized by multiple media scholars for distributing the Western culture across the world and working as a vehicle of colonialism (Mayer et al. 2022; Kwon et al. 2022). There is a need to take stock of these important critiques when considering the visual culture facilitated by GenAI technology. It is important to note, that none of the dynamics described above are unique to AI-generated imagery—but rather they are found in visual culture and the specific mediums that convey it, such as vGenAI in today’s world and in the future. As for the systemic nature of the issues, there is no quick fix for solving them.

Our intersectional visual analysis (particularly the insights found through analyzing the institutional embeddedness of SD) also further reinforces the need for appropriate regulation of these rapidly growing and evolving technologies, which pays attention to the social inequalities they mirror and perpetuate, takes seriously the concerns around the use and recreation of harmful and violent images, and assigns criminal culpability when appropriate. It also shows the need to continually fight for more diversity in the teams developing and maintaining such technical systems, in the hope of addressing some of the inequalities and exclusions expressed through these images (Kapania et al. 2023). However, such recommendations do not go far enough given the attention towards institutional dynamics and prevalent power systems like racism, colonialism, and capitalism, which our intersectional approach highlighted. Thus, we structure our recommendations around Davis et al. (2021)’s concept of ‘algorithmic reparation’ which they propose as ‘a foundation for building, evaluating, adjusting, and when necessary, omitting and eradicating machine learning systems’ which itself is rooted in intersectional theory and approaches. This reparative approach focuses on the offending parties symbolically and materially mending wrongdoings enacted against individuals and groups (ibid.). This may involve literal reparation payments, but may also be more about acknowledging and seeking to address identified harms. One major harm that we identified in the case of SD was the perpetuation and magnification of stereotypes, prejudices, and exclusions. A reparative approach would advocate for more research on the various impacts of the images on the subjectivities of marginalized individuals who are exposed to this imagery (e.g. Zhou and Nabus 2023) in order to understand both how this can be mitigated but also what support or resources should be given to those affected. It would also advocate for deliberative intervention in vGenAI systems in order to address these inequalities and exclusions, counter to the prevailing ‘algorithmic idealism’ (Davis et al. 2021) of AI developers which assumes that human intervention must be minimized to retain ‘objectivity’. Such interventions could include efforts to curate training data better, and involve designers who have the explicit responsibility to study and identify better ways of designing and configuring the visual aesthetics of these systems, using an intersectional approach and algorithmic reparation. Davis et al. (2021) also describe how in the case of other Machine Learning systems the general approach is to invisibilize social categories such as gender, race, and sexuality in organizing systems on the assumption that this creates more ‘neutral’ results. However, again, a reparative approach would advocate for a more explicit acknowledgment of these categories to acknowledge, make visible, and address inequalities. Hence, we advocate rendering visible the visual politics of portrayal (Jääskeläinen et al. 2024) to acknowledge the vGenAI imagery’s cultural-political embeddedness, and extending this critical outlook into problematizing the very core notions of vGenAI technology.

A much broader harm which we have identified in this paper resulting from imagery produced through SD and other vGenAI tools is in its impacts on global visual culture through the sheer quantity of images that can be produced and quickly circulated. This raises concerns about the homogenization of global visual culture along the lines of the photorealist style which is mimicked by the main vGenAI providers, but also in terms of the broader values, inequalities and exclusions which are encoded in these images. A reparative approach here would be to see regulation not only as a tool to shape the development of vGenAI but also to proactively limit its power in the name of retaining a greater diversity of visual styles and different forms of image creation. This also shows that AI developers must bear some responsibility towards artists and other creatives who are likely to lose business due to the ease of use and cheapness of vGenAI tools, which may be formalized through grants and other forms of financial support. Users of vGenAI (such as artists and creators) can also contribute to a more reparative approach by being more conscious of how they are generating images—e.g. what kind of prompts they use, and what kind of aesthetics they pick into the images that are used in their work and further distributed in the society. However, it is clear that the value-laden aesthetics position artists and creators differently in relation to the vGenAI technology. For example, it might benefit artists of Western origin that do not primarily practice critical image-making and work in a capitalist setting with a demand for fast-paced workflows, whereas some artists might lose their work opportunities and creative capital due to the visual politics of the system and appropriation of their work. Therefore, the impact of AI on artists and creative practitioners must be examined from an intersectional perspective to fully understand the impact on social categories and subjectivities. It is well known that the creative industries already exhibit deep inequalities, with white, upper-class males perceived as securing more lucrative employment. We must therefore also examine the impacts of the vGenAI technology when it comes to various identities of artists; white artists and colored artists, artists based in various contexts of the Global North and the Global South, trans- and gender non-confirming artists and their cis-counterparts. While artists and creatives often invest years honing their craft and developing a unique artistic style, the unauthorized use of their work or its replacement by synthetic art can cause significant financial detriment, especially given the challenges many artists already face (Sætra 2023). Despite these notable criticisms, Stability AI founder Mostaque has furthermore accused artists of seeking a ‘monopoly’ on visual communication. This critique could be perhaps taken seriously if the vGenAI companies would responsibly hire artists and image-makers to get paid to produce their training data, or exclusively use free-license imagery. But unfortunately, companies deeply embedded in capitalist profit-seeking have been repeatedly demonstrated that they do not see a problem in exploiting artists as stakeholders in an attempt to gain market advantage. While exploiting artists' work, the tech companies often simultaneously approach copyright violations in vastly different way when it comes to their own technology. For example, with the recent allegations of Deepseek 'distilling' OpenAI models, the company warned: "we take aggressive, proactive countermeasures to protect our technology and will continue working closely with the US government to protect the most capable models being built here” (Reed, 2025). This signals a certain hypocracy and a vastly different attitude towards labor and intellectual property of creatives and technology companies.

Lastly, it is notable that AI tools monopolizing available artistic tools can have significant impacts on aesthetics and everyday creative practices. Artists who are using vGenAI need more knowledge and resources that can inform the critical use of these tools. With all the problems embedded in SDXL and other vGenAI tools, artists can easily become vehicles for redistributing the neo-colonizing imagery in society through uncritical creative practice. This further highlights the need for diversifying the visual aesthetics of vGenAI models, but also supporting creatives in taking a critical outlook when using vGenAI technologies. While our study contributes to developing such knowledge, further work can examine how such intersectional and justice-oriented (Costanza-Chock 2018, 2020), and algorithmic reparation perspectives can be brought into the socio-technical and visual cultural–political landscape of vGenAI in everyday practices.

## Data Availability

All images and prompts used to produce the intersectional analysis will be available upon request to primary authors.

## Footnotes

1 Although the focus is on SD specifically, the paper at times utilizes general critiques about vGenAI. It is important to highlight that while many general critiques apply to SD, the underlying algorithms, training data sets, and ML models are different for each specific technology. We aim for caution in being as specific as possible when making claims, and urge the readers to be mindful of this difference.

2 By simplistically interpreting the images using terms such as ‘man’ and ‘woman’, we are at danger of reinforcing the binary gender norm (the assumption that people of a certain gender should be associated with a certain set of visual features). Thus, throughout this paper we use the terms "hetero-normative feminine/masculine representations" in discussing heteronormative gender norms in visual aesthetics.

3 This research underwent ethical review and approval by the institutions affiliated with the two main authors.
